# Immunometabolic function in depression: From etiology to treatment

**DOI:** 10.1192/j.eurpsy.2025.163

**Published:** 2025-08-26

**Authors:** B. Penninx

**Affiliations:** psychiatry, Amsterdam UMC, Amsterdam, Netherlands

## Abstract

**Abstract:**

**Background:**

Major depressive disorder is a common, disabling mental disorder characterized by extensive etiological and phenotypic heterogeneity. This heterogeneity makes treatment approaches imprecise and often ineffective. Insight into the underlying biological mechanisms underpinning depression and its subtypes may enable more personalized treatments.

**Methods:**

A review of the literature as well as data analyses from 2981 individuals from the Netherlands Study of Depression and Anxiety (NESDA), of whom ˜1900 persons have or lifetime or current Major Depressive Disorder and 650 were healthy controls.

**Results:**

Significant immuno-metabolic dysregulations are present in about 20-30% of people with depression. Such immuno-metabolic depression is characterized by the clustering of 1) atypical, energy-related depressive symptoms such as hypersomnia, fatigue, hyperphagia, and possibly anhedonia, 2) systemic low-grade inflammation with elevated levels of e.g. C-reactive protein, cytokines and glycoprotein acetyls, and 3) metabolic abnormalities involving e.g. obesity, dyslipidaemia, insulin and leptin resistance. Evidence for such clustering is confirmed in large-scale proteomic, metabolomic, gene expression as well as genome-wide data analyses. Persons with immuno-metabolic depression are at a higher risk for cardiometabolic diseases and – from pooled analyses of 4 RCTs in over 1000 individuals - seem to respond less well to standard antidepressant treatment.

**Discussion:**

Interventions targeting inflammation, metabolism or lifestyle may be more effective treatment options for individuals with immuno-metabolic depression, in line with principles of precision psychiatry.

**Disclosure of Interest:**

None Declared

